# Effect of mushroom *Agaricus blazei* on immune response and development of experimental cerebral malaria

**DOI:** 10.1186/s12936-015-0832-y

**Published:** 2015-08-11

**Authors:** Cynthia H Val, Fátima Brant, Aline S Miranda, Flávia G Rodrigues, Bruno C L Oliveira, Elândia A Santos, Diego R R Assis, Lísia Esper, Bruno C Silva, Milene A Rachid, Herbert B Tanowitz, Antônio L Teixeira, Mauro M Teixeira, Wiliam C B Régis, Fabiana S Machado

**Affiliations:** Department of Biochemistry and Immunology, Institute of Biological Science, Federal University of Minas Gerais, Bloco O4, 190 Av. Antônio Carlos, 6627-Pampulha, Belo Horizonte, MG 31270-901 Brazil; Programme in Health Sciences: Infectious Diseases and Tropical Medicine, Medical School, Federal University of Minas Gerais, Belo Horizonte, MG Brazil; Programa de Pós Graduação em Biologia de Vertebrados da Pontifícia Universidade Católica de Minas Gerais, Belo Horizonte, MG Brazil; Department of Pathology, Institute of Biological Science, Federal University of Minas Gerais, Belo Horizonte, MG Brazil; Department of Pathology and Department of Medicine, Albert Einstein College of Medicine, Bronx, NY USA

**Keywords:** *Agaricus blazei* Murrill, Experimental cerebral malaria, Immunomodulation, Anti-malarial therapy

## Abstract

**Background:**

Cerebral malaria (CM) is debilitating and sometimes fatal. Disease severity has been associated with poor treatment access, therapeutic complexity and drug resistance and, thus, alternative therapies are increasingly necessary. In this study, the effect of the administration of *Agaricus blazei*, a mushroom of Brazilian origin in a model of CM caused by *Plasmodium berghei,* strain ANKA, was investigated in mice.

**Methods:**

C57BL/6 mice were pre-treated with aqueous extract or fractions of *A. blazei*, or chloroquine, infected with *P. berghei* ANKA and then followed by daily administration of *A. blazei* or chloroquine. Parasitaemia, body weight, survival and clinical signs of the disease were evaluated periodically. The concentration of pro-and anti-inflammatory cytokines, histopathology and in vitro analyses were performed.

**Results:**

Mice treated with *A. blazei* aqueous extract or fraction C, that shows antioxidant activity, displayed lower parasitaemia, increased survival, reduced weight loss and protection against the development of CM. The administration of *A. blazei* resulted in reduced levels of TNF, IL-1β and IL-6 production when compared to untreated *P. berghei*-infected mice. *Agaricus blazei* (aqueous extract or fraction C) treated infected mice displayed reduction of brain lesions. Although chloroquine treatment reduced parasitaemia, there was increased production of proinflammatory cytokines and damage in the CNS not observed with *A. blazei* treatment. Moreover, the in vitro pretreatment of infected erythrocytes followed by in vivo infection resulted in lower parasitaemia, increased survival, and little evidence of clinical signs of disease.

**Conclusions:**

This study strongly suggests that the administration of *A. blazei* (aqueous extract or fraction C) was effective in improving the consequences of CM in mice and may provide novel therapeutic strategies.

**Electronic supplementary material:**

The online version of this article (doi:10.1186/s12936-015-0832-y) contains supplementary material, which is available to authorized users.

## Background

There is a rich history of the use of natural products in the treatment of parasitic diseases, including malaria. Mushrooms have been used for nutritional and medicinal purposes since ancient times [1]. The mushroom *Agaricus blazei* is of Brazilian origin and has been used as a functional food and a popular medicine [2]. Many laboratories have investigated the effect of *A. blazei* bioactive components as an antioxidant [3–5] and as immunomodulatory agents [6, 7]. There is increased interest in these compounds in various disease states, such as cancer [7–11], allergy [12, 13], inflammatory diseases [13, 14], viral and bacterial infections [6, 7, 15], diabetes [16, 17] and cholesterol biosynthesis [16]. Furthermore, there has been great interest in the use of this mushroom in the treatment of leishmaniasis [18–20]. The use of this mushroom in the treatment of malaria has not been evaluated.

Malaria, caused by *Plasmodium* species remains an important cause of morbidity and mortality especially in Africa, parts of Asia and Latin America. Malaria accounts for millions of deaths and many more millions are at risk. Infections caused by *Plasmodium falciparum* are often the most severe [21]. The most debilitating phenotype of severe malaria is a neurological syndrome known as cerebral malaria (CM) [22–24], which predominantly affects children in sub-Saharan Africa. This disease has a mortality rate around 20 % and is accompanied by seizures and neurocognitive dysfunction [21, 25]. The treatment of CM in endemic areas is complicated due to lack of access and cost of drugs and the development of resistance [23, 26–29]. Additionally, as noted successful anti-malarial therapy is effective in eliminating the parasite from the bloodstream, but does not prevent the development of neuronal damage [26, 28, 29].

*Agaricus blazei,* or some of its fractions, stimulates the immune response, including TNF and IL-8 production by macrophages [30] and stimulates IL-1β and IL-6 in human monocytes and endothelial cells [6]. Studies in healthy individuals fed *A. blazei* demonstrated significant reduction in cytokine levels including TNF, IL-1β, IL-2, IL-6, and IL-17 in human blood [31]. Blood cells obtained from patients, with inflammatory bowel disease treated with *A. blazei* had reduced levels of IL-1β, IL-6, IL-8, MCP-1 and G-CSF when stimulated with LPS in vitro [14]. Johnson et al. [31] observed in human healthy volunteers, after oral *A. blazei*, elevated levels of, IFN-γ, IL-2, IL-4, IL-10, IL-12 and IL-17 in blood/serum.

An aqueous extract of *A. blazei* reduced the parasite infectivity, load and viability [18] in murine macrophages infected with different species of *Leishmania*. Additionally, *Leishmania amazonensis*-infected mice treated with aqueous extract of *A. blazei* displayed a reduction of the lesions size, a reduction of parasite load in the spleen and lymph nodes, an elevation of IFN-γ and decreased IL-4 and IL-10 in the spleen and in lymphoid nodules, respectively [19]. *A. blazei* has also been used in the prophylaxis and treatment of *Leishmania chagasi* infection [20].

Herein, there is evidence that *A. blazei* has antioxidant activity and that *A. blazei* extract or a purified fraction inhibited the production of pro-inflammatory cytokines in the brain (TNF, IL-1β and IL-6) and spleen (IFN-γ, IL-6 and IL-17) during *Plasmodium berghei* (strain ANKA) infection preventing the development of severe disease. Treatment with chloroquine resulted in an increased production of most pro-inflammatory cytokines analyzed in the brain when compared to untreated-infected and *A. blazei*-treated infected mice. We also demonstrated that *A. blazei* and chloroquine administration decreased parasitaemia, increased survival, but *A. blazei* demonstrated greater protection against brain damage when compared with chloroquine.

## Methods

### Preparation of the aqueous extract and fractions purified of the mushroom *Agaricus blazei*

*Agaricus blazei* aqueous extract was prepared by mixing 5 g of the mushroom powder form with 50 mL of milli-Q water at a concentration of 1.5 % (weight/volume) for a 2 h incubation at room temperature, followed by centrifugation at 7,800×*g* (Thermo Scientific Heraus Multifuge X1R) at 4 °C for 30 min. The supernatant was obtained (crude aqueous extract) and subjected to lyophilization (Lyophilizer LIOTOP, K105) at a temperature of −101 °C and a pressure of 23 mmHg for 48 h. At the end of this process a product was obtained (crude lyophilized extract). The aqueous extract underwent centrifugation at 2,000×*g* for 45 min at 4 °C using different columns (to give three fractions with different molecular weights). The samples were subjected to the same process lyophilizing as above and then it were stored at −20 °C, until use. This procedure has been patented at CT&T/UFMG.

### Chemical profile

For the determination of all chemical compounds present in *A. blazei*, the lyophilized samples were resuspended in advance at a concentration of 1 mg/mL. All tests were performed in triplicate.

#### Protein determination by the Lowry method

Protein determinations of *A. blazei* extracts and fractions were performed with aliquots of 30 μL of each sample by adding 170 μL of Milli-Q water and 2.1 mL of biuret (Synth, Diadema, São Paulo, Brazil). After 10 min in the dark, 200 μL of a solution of Folin–Ciocalteau (1:2) (Sigma-Aldrich, St. Louis, MO, USA) was added, samples were then homogenized by vortex (Cyclo mixer—Clay Adams) and left to stand in the dark for 1 h. Absorbance was then measured at 750 nm in a spectrophotometer (Shimaduzu, UV-160A). Milli-Q water was used as a negative control following the same pattern of samples containing biuret solution and Folin–Ciocalteau solution. A calibration curve was constructed with different concentrations of bovine serum albumin (BSA) (Sigma-Aldrich, St. Louis, MO, USA) for comparison.

#### Determination of phenolic compounds

The determination of phenol compounds in *A. blazei* mushroom Fractions and extract was performed using 80 μL aliquot of each sample. For analysis we added 320 μL of methanol (Sigma-Aldrich, St. Louis, MO, USA), 250 μL of Folin–Ciocalteau (Sigma-Aldrich, St. Louis, MO, USA), 3 mL of Milli-Q water and 1 ml of sodium carbonate 15 % (w/v) (Synth, Diadema, São Paulo, Brazil). Homogenization was performed in shaker (Cyclo mixer—Clay Adams), the samples were kept in the dark for 1 h. Afterwards, absorbance was determined at 750 nm in a spectrophotometer (Shimaduzu, UV-160A). As a negative control, enzymatic reaction was used, 80 μL of methanol following the same pattern of samples containing Folin–Ciocalteau reagent (Sigma-Aldrich, St. Louis, MO, USA), Milli-Q water and sodium carbonate 15 % (w/v) (Synth, Diadema, São Paulo, Brazil). Samples were then compared to a calibration curve of different concentrations of gallic acid (Riedel-de Haën) 0.5 mg/mL.

#### Determination of flavonoids

To an aliquot of 200 μL we added 800 μL of ethanol, to samples and then its was diluted with 150 μL of sodium carbonate 10.6 %, 300 μL of aluminum chloride in 2 % methanol (all reagents from Synth, Diadema, São Paulo, Brazil), 1 mL of sodium hydroxide 1 M (Merck KGaA, Darmstadt, Germany) and 1 mL of Milli-Q water. After 5 min of incubation, the absorbance was read at 410 nm in a spectrophotometer (Shimaduzu, UV-160A). As a negative control, enzymatic reaction was used, 1 mL of Milli-Q water, 800 μL of ethanol (Synth, Diadema, São Paulo, Brazil), sodium carbonate 10.6 %, aluminum chloride 2 % and 1 mL of sodium hydroxide (Sigma-Aldrich, St. Louis, MO, USA) for comparison. Subsequently, the percentage of flavonoids was calculated in the total sample weight.

#### Determination of carbohydrates

Samples were diluted (50×), and 200 μL aliquots stored until further use. 200 μL of phenol solution (5 %) (Merck KGaA, Darmstadt, Germany) were added to the sample, followed by 1 ml of sulfuric acid (Sigma-Aldrich, St. Louis, MO, USA). The homogenization was performed in shaker (Cyclo mixer—Clay Adams) and after 15 min, the absorbance was read at 480 nm using a UV–VIS spectrophotometer (Shimaduzu, UV-160A). As a negative control we used 200 μL of reaction solution, Milli-Q water, 200 μL of phenol reagent and 1 ml of sulfuric acid (all reagents from Merck KGaA, Darmstadt, Germany). A calibration curve with standard solutions of glucose was constructed for comparison and calculation of the percentage in the total sample weight.

### Test of antioxidant activity

Antioxidant activity was determined by the method of DPPH (2,2-diphenyl-1-picryl-hydrazyl) (Sigma-Aldrich, St. Louis, MO, USA) proposed by Brand-Williams et al. [32]. Briefly, to 5 µL of sample we added 195 µL of DPPH solution (60 µM). The absorbance reading was performed in a spectrophotometer at 515 nm (Varioskan Flash, Thermo Scientific) until its stabilization. The maximum antioxidant activity was calculated by converting the absorbance percentage in antioxidant activity (AA maximum) using the formula: AA maximum (%) = 100 − {[(Abs sample − Abs blank) × 100]/Abs control}. The negative control was prepared with 5 µL of methanol solution (50 %) and 195 µL of DPPH. The decrease in absorbance represents stabilization of this free radical.

### Polyacrylamide gel electrophoresis

Samples of *A. blazei* extract or fractions were subjected to PAGE in the presence of SDS [33]. Briefly, electrophoretic run was performed in a vertical electrophoresis containing running buffer (0.025 M Tris–HCl, pH 8.3, 0.1 % SDS) under constant voltage (100 V) for 3 h and 30 min. After migration, the protein in the gel was stained according to the silver method staining and the gel washed with Milli-Q water five times for 5 min each wash. After 2 h in the pre-fixative A (solution 50 % methanol and 7.5 % acetic acid), there was an additional of 2 h incubation in pre-fixative B (solution 5 % methanol and 7.5 % acid acetic acid).

### Administration of extracts or fractions of *Agaricus blazei*

Wild-type (WT) C57BL/6 females (8–10 weeks old) mice were obtained from the Animal Care Facilities of Federal University of Minas Gerais, Belo Horizonte, Brazil. C57BL/6 mice females (n = 5 mice/group) received by gavage (3 days before infection) 250 μL daily of crude extract or Fractions of *A. blazei* or chloroquine—hydroxychloroquine sulfate (Aspen Pharma, Johannesburg, South Africa, antimalarial drug used as control in the experiments). Doses used were as follows: aqueous extract: 10 and 100 mg/kg of body weight; lyophilized extract: 80 and 800 mg/kg of weight; fraction A: 10 mg/kg of weight; fraction B: 10, 80 and 800 mg/kg of weight; fraction C: 100 and 800 mg/kg of weight; chloroquine: 30 mg/kg of weight. Dosages were based on the weight of 25 g/animal. Extracts and fractions were diluted and/or suspended in Milli-Q water in order to keep the concentration and dose similar. After pretreatment with crude extract or fractions of *A. blazei* or chloroquine for 3 days, mice were infected with *P. berghei* (strain ANKA), followed by continuous administration of *A. blazei* or chloroquine until 7 dpi. Experiments were performed on day 5 post-infection (dpi) when *P. berghei*-infected mice develop brain inflammation without motor impairment [34, 35]. This study was carried out in strict accordance with the Brazilian Guidelines on animal work and the Guide for the Care and Use of Laboratory Animals of the National Institutes of Health. The animal ethics committee of the Universidade Federal de Minas Gerais CETEA/UFMG approved all experiments and procedures including euthanasia, fluid and organ removal (Permit Number: 262/11).

### Parasitology and experimental infection

Blood stages of *P. berghei* constitutively expressing green fluorescent protein (*P. berghei* ANKA-GFP) (15cy1 clone) [24], kindly provided by Dr. Claudio Marinho (University of São Paulo), were stored in liquid nitrogen. Mice were infected intraperitoneally (i.p.) with 10^5^*P. berghei*-infected red blood cells suspended in 0.2 mL PBS. The percentage of parasitaemia was quantified by GFP frequency in whole blood using flow cytometry according to the methodology described by Brant et al. [22].

### Parasitaemia, clinical signs and liver function

Mice were observed daily for parasitaemia, survival and clinical signs of CM (i.e. ataxia, paralysis, and coma). CM signs were evaluated using the rapid murine coma and behaviour scale (RMCBS), a protocol based on the components of the SHIRPA (SmithKline/Harwell/Imperial College/Royal Hospital/Phenotype Assessment) score [36]. The RMCBS consists of ten parameters (gait, balance, motor performance, body position, limb strength, touch scape, pinna reflex, toe pinch, aggression and grooming), and each item is scored from zero as the lowest, to two as the highest, with a maximum total score of 20. This scale is a quantitative and objective method that enables a rapid follow up of CM course. This assessment was carried out daily from day 3 dpi until day 7 dpi. For haematocrit determination at 5 dpi, blood samples were collected into heparinized capillary tubes, and centrifuged for 10 min in a haematocrit centrifuge (HT, São Paulo, Brazil). Liver function of *P. berghei*-infected and control mice was determined by measuring the levels of alanine aminotransferase (ALT) and aspartate aminotransferase (AST) in serum at 5 dpi using a commercially available kit following the manufacturer’s protocol (Quibasa, Bioclin, Belo Horizonte, Brazil).

### Brain pathology and quantification of cytokines

At 5 dpi mice were euthanized with ketamine/xylazine, the brain was carefully removed, immediately fixed in 4 % buffered formalin and tissue fragment was embedded in paraffin. Tissue sections (4 µm thick) were stained with haematoxylin and eosin (H&E) and examined under light microscopy. Sections were captured with a digital camera (DEI-470; Optronics, Goleta, CA, USA) connected to a microscope (IX70; Olympus, Center Valley, PA, USA) with a magnification of 20×. For quantification of cytokines, brains and spleens were homogenized in a PBS-buffer containing a protease-inhibitor cocktail to determine the concentrations of the cytokines IFN-γ, TNF, IL-1β, IL-6, IL-10, IL-12, IL-17 and TGF-β by enzyme-linked immunosorbent assay (ELISA) DuoSet kits (R&D Systems, Minneapolis, MN, USA), in accordance to the manufacturer’s instructions.

### Treatment of infected red blood cells in vitro

Parasitized blood was collected from *P. berghei*-infected mice displaying a 5–8 % parasitaemia and was pretreated with a concentration of 500 μg/mL of *A. blazei* aqueous extract, lyophilized extract or fraction C. After 1 h of incubation, untreated infected red blood cells or *A. blazei*-treated infected red blood cells were centrifuged (Centrifuge 5415R, Eppendorf) in 100×*g* for 5 min at 24 °C. The supernatant was discarded and PBS was added, followed by intraperitoneal inoculation in C57BL/6 mice.

### Statistical analysis

Data was analysed by statistical and Graph Prism Software 4.0 (GraphPad, La Jolla, CA, USA). Results are shown as mean ± standard error of the mean (SEM). Differences were compared by using two-tailed Student’s t tests with 95 % confidence intervals, analysis of variance (ANOVA) or Two-way ANOVA followed by Bonferroni’s corrections as needed for multiple comparisons when parametric assumptions were met. Differences between lethality curves were calculated using Log rank test. Results with a P < 0.05 were considered significant.

## Results

### Characterization of the *Agaricus blazei* crude extract

**Fig. 1:**
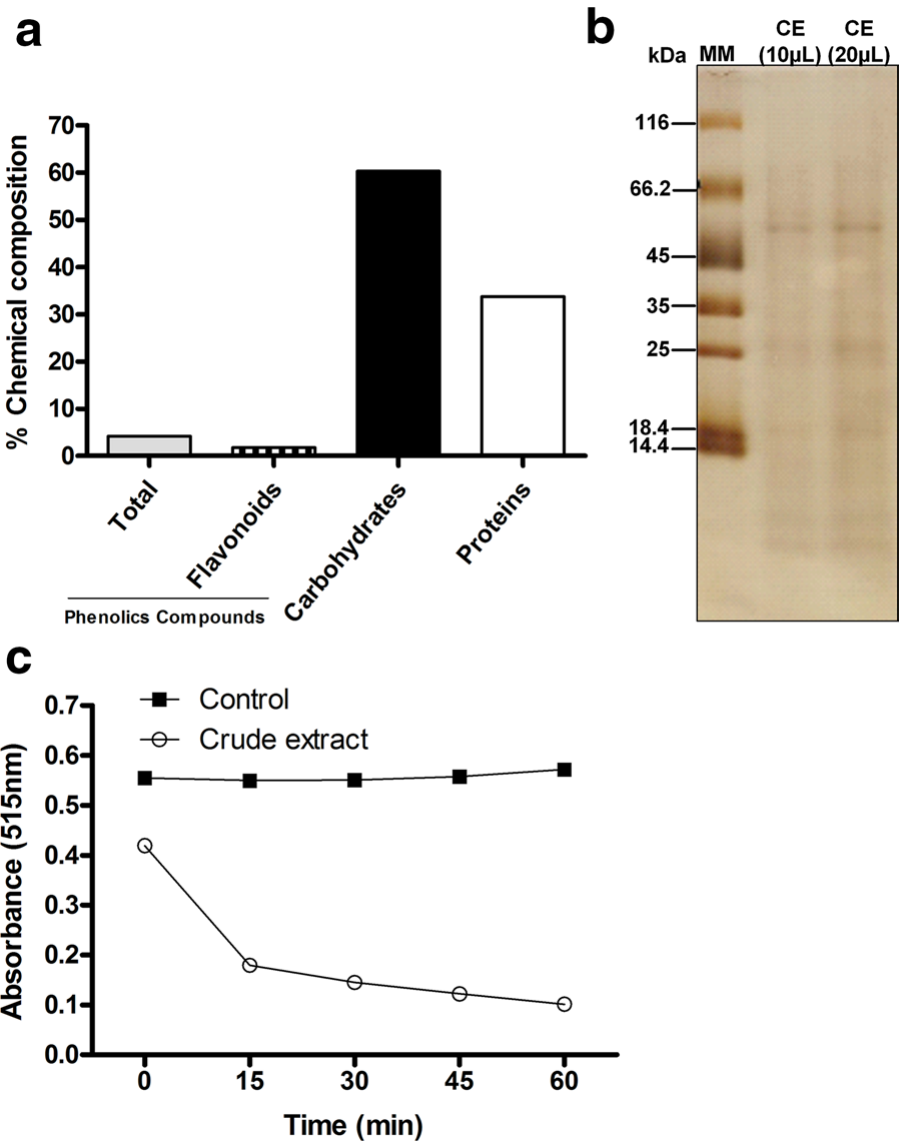
Characterization of the crude extract of *Agaricus blazei*. **a** Partial chemical composition of the crude extract of *A. blazei*. **b** Analysis by SDS-Page 12 % polyacrylamide of the crude extract of *A. blazei*. **c** Measurement of antioxidant activity of the crude extract from the absorbance of DPPH free radical, the decrease in absorbance represents stabilization of this free radical. *MM* molecular mass markers, *CE* crude extract of *A. blazei.*

The chemical characteristics of the *A. blazei* crude extract were investigated. Figure 1 demonstrates that the *A. blazei* crude extract is rich in carbohydrates, proteins and, to a lesser extent, phenolic compounds (Fig. 1a). The protein profile ranged from 45 to 25 kDa (Fig. 1b) and its concentration was also used as a reference for the analyses of the anti-malarial activity (based on previous studies of the microbicidal activity of *A. blazei* against *Leishmania* infection [18–20]. Figure 1c, shows that this extract has elevated antioxidant capacity.

### Administration of *Agaricus blazei* aqueous extract decreases parasitaemia and increases survival of *Plasmodium berghei*-infected mice

**Fig. 2:**
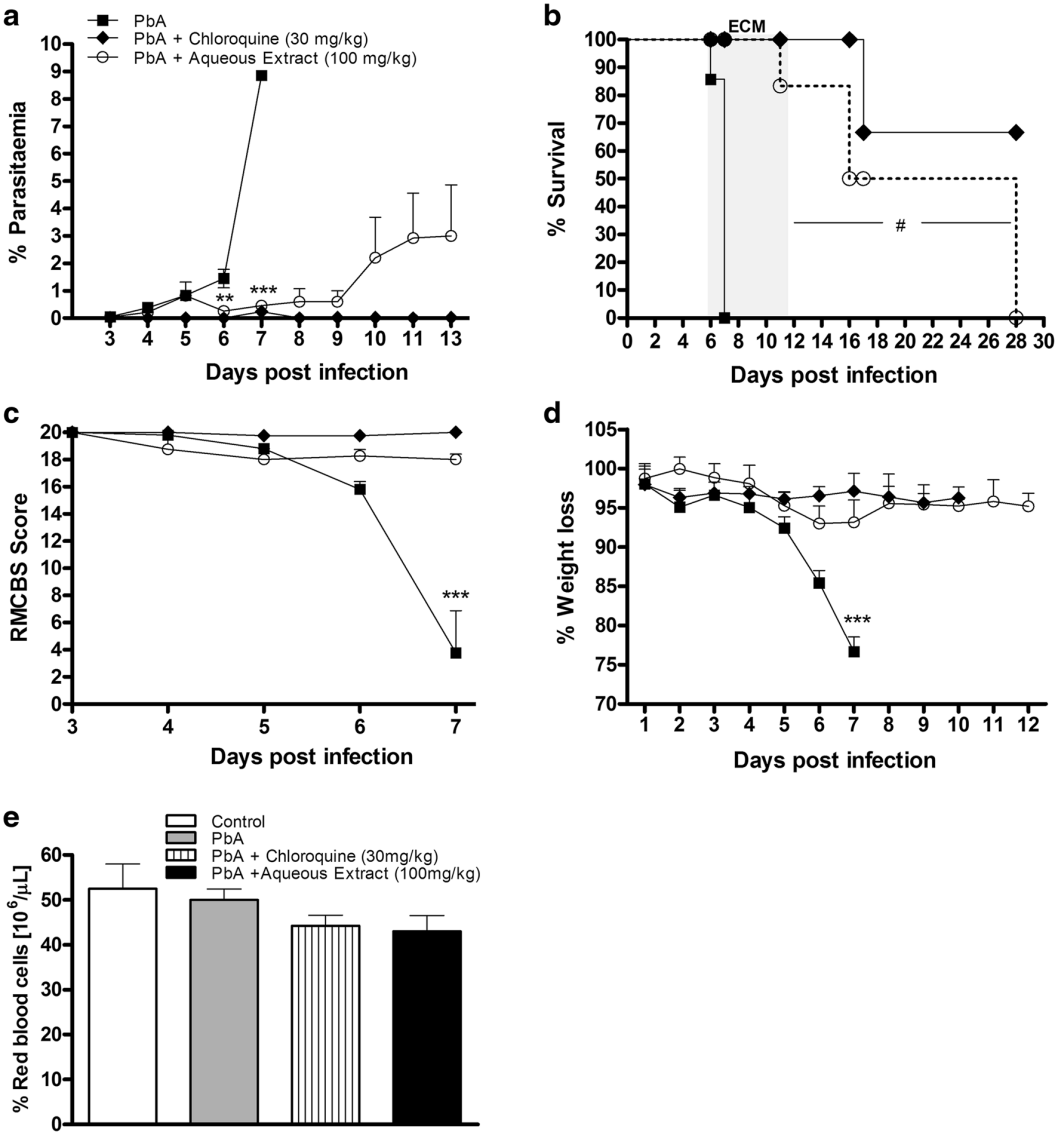
Administration of aqueous extract results in the resistance of mice to *Plasmodium berghei*-infection. C57BL/6 mice received 100 mg/kg of *A. blazei* extract or chloroquine (30 mg/kg) 3 days before infection, then infected with 10^5^ *P. berghei* red blood cells (pRBCs), and treated until 7 dpi. **a** Parasitaemia, **b** survival and **c** clinical signs of cerebral malaria assessed by the RMCBS scale and **d** body weight of untreated *P. berghei*-infected mice and treated with chloroquine or *A. blazei* aqueous extract. Neurologic signs of CM appeared on days 6–12 (*shaded area*), with death occurring 24–48 h after onset. **e** Haematocrit was assessed on 5th dpi in the above groups and in mice uninfected. Parasitaemia, body weight and haematocrit (%) values are expressed as mean ± SD of 5 mice per group. ^#^ *p* < 0.001, log-rank test and **p < 0.01 and ***p < 0.001, ANOVA followed Bonferroni’s test.

*Agaricus blazei* aqueous extract-treated mice (100 mg/Kg) displayed a significant decline in parasitaemia when compared with untreated mice at 6 and 7 dpi (Fig. 2a). There was increased survival (~85 %) for an additional 20 days (Fig. 2b), and no clinical signs were noted (Fig. 2c). Untreated *P. berghei*-infected mice (~90 %) developed ataxia, loss of grip strength, progressive paralysis, coma, ruffled fur and significant weight loss (Fig. 2c, d) between 6 and 7 dpi. *A. blazei* aqueous extract-treated mice had non-significant reduction in body weight (Fig. 2d). None of the groups showed significant differences in haematocrit at 5 dpi (Fig. 2e). When *A. blazei* aqueous extract was administrated to infected mice at dose of 10 mg/kg the course of infection was comparable with untreated infected mice (see Additional file 1A-C) indicating, that in low concentration this extract is not effective. Next, was then tested whether the *A. blazei* extract in lyophilized form could exert anti-malarial activity since lyophilized formulations increase storage time and the product quality [37]. Administration of 80 mg/kg of *A. blazei* lyophilized extract did not confer resistance against *P. berghei*-infection (see Additional file 2A, B). However, administration of 800 mg/kg of *A. blazei* lyophilized extract to infected mice resulted in significant survival (~80 %) and no visible clinical sings of CM (see Additional file 2A, B). Transaminase determinations revealed no hepatic cytotoxic effects during *A. blazei* administration (see Additional file 3A, B). The mushroom extract has very low toxicity in animal models [38, 39] and is likely safe for human at concentrations from 900 to 1,500 mg/day for until 1 year of treatment [40–42]. Mice treated with chloroquine displayed a decline in parasitaemia and increased survival (Fig. 2).

### Administration of purified *Agaricus blazei* fraction protects against *Plasmodium berghei* infection

**Fig. 3:**
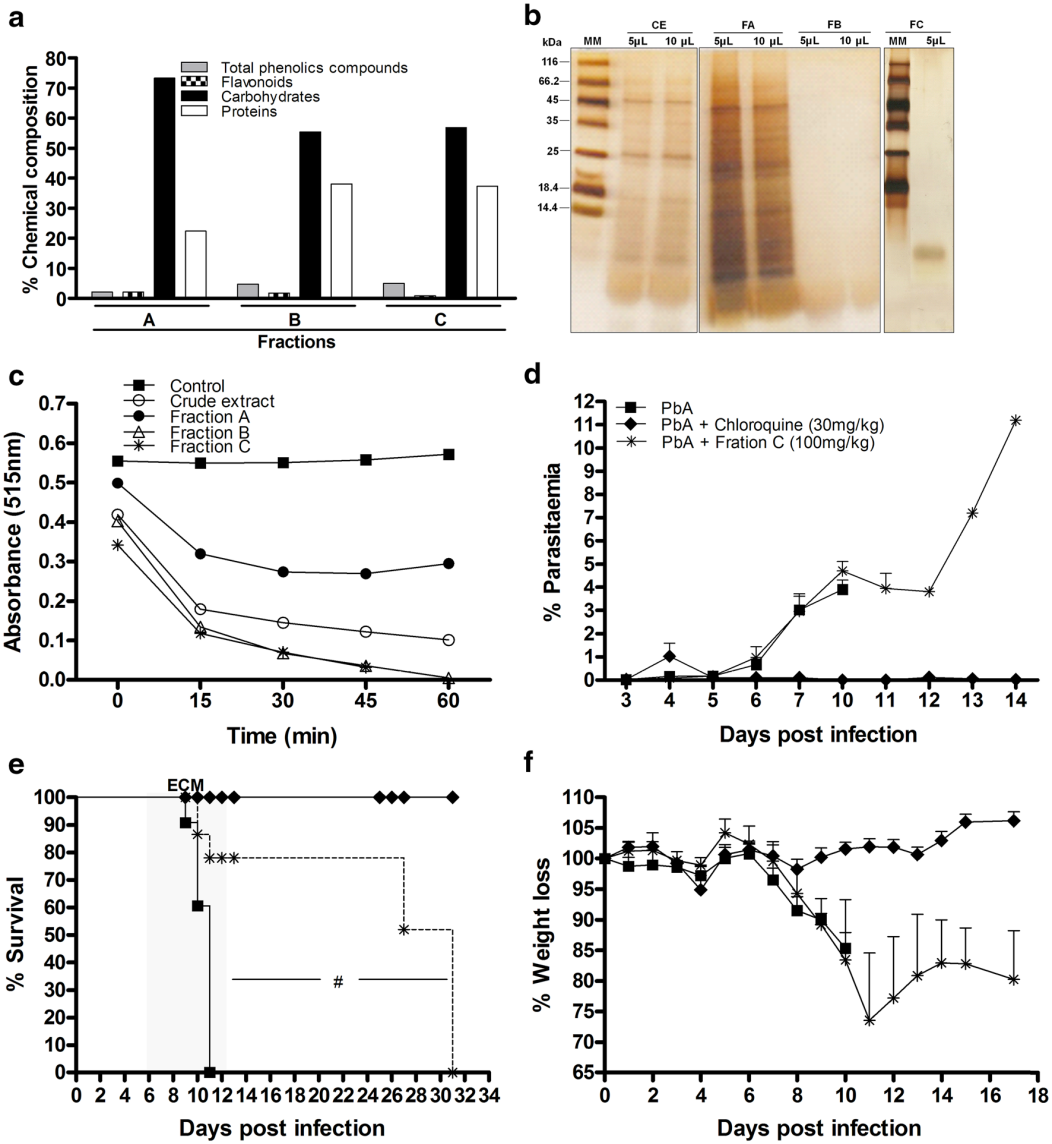
Administration of fraction C of *Agaricus blazei* protects mice against *P. berghei* infection. **a** Characterization of fractions of the crude extract of *A. blazei* with the partial chemical composition of fractions A–C (in percentage), **b** analysis by SDS-Page 12 % polyacrylamide of fractions obtained from the purification of the crude extract and **c** measurement of antioxidant activity of fractions of AbM. *MM* molecular mass markers, *CE* Crude Extract, *FA* Fraction A; *FB* Fraction B; *FC* Fraction C. C57BL/6 mice received 100 mg/kg of *A. blazei* Fraction C or chloroquine (30 mg/kg) 3 days before infection, then infected with 10^5^ parasitized RBCs IP via, and treated until 7th day post infection. **d** Parasitaemia, survival and body weight in untreated *P. berghei*-infected mice and treated with chloroquine or fraction C. Neurologic signs of CM appeared on days 6–12 (*shaded area*), with death occurring 24–48 h after onset. Parasitaemia and body weight values are expressed as mean ± SD of 5 mice per group. ^#^ *p* < 0.001, log-rank test; ANOVA followed Bonferroni’s test.

Next was determined whether there was a specific *A. blazei* purified fraction responsible for the observed anti-malarial activity. It was found fractions A, B and C, which were characterized by chemical composition, protein profile and antioxidant activity (Fig. 3a–c), were separated by differences in MW. Fraction A had a MW greater than 10 kDa, the MW of fraction B was between 3 and 10 kDa, and fraction C had a MW less than 3 kDa. All three fractions had large amounts of carbohydrate (73.37 % for fraction A, 55.37 % for fraction B and 56.86 % for fraction C). Fractions B and C contained higher levels of protein (38.11 and 37.37 %, respectively) and phenolic compounds (4.73 and 4.96 %, respectively) than fraction A (22.39 % protein and 2.12 % phenolic compounds). Fraction C contained the lowest percentage of flavonoids (0.81 %) (Fig. 3a). The protein profile demonstrated several proteins with different MWs. However, both fractions B and C had proteins with MWs less than 14.4 kDa (Fig. 3b). The high protein content of the fractions B and C is likely due to the presence of peptides (Fig. 3b). There were higher antioxidant activities in fractions B and C, and lower activity in fraction A (Fig. 3c).

The anti-malarial activity of these three fractions was also evaluated. There were no significant differences in parasitaemia and mortality between those mice treated with fraction A (10 mg/kg) and untreated infected mice (see Additional file 4A-C), and this fraction was no longer studied. The fraction B was administered at doses of 10, 80 and 800 mg/kg (see Additional file 5). It was observed that different doses of fraction B were not effective in controlling parasite replication, and treated mice died between 6 and 10 dpi, similar to untreated infected mice (see Additional file 5A, B). Next, the anti-malarial activity of fraction C was examined using a dose of 100 mg/kg (Fig. 3d), the same used for the aqueous extract. Mice treated with fraction C did not show significant differences in parasitaemia during the first 7 dpi, contrasting the results observed with aqueous *A. blazei* treatment. Despite this, fraction C-treated-mice survived more than 15 dpi (~80 %) (Fig. 3e) and did not display clinical signs (see Additional file 6A) of CM when compared to untreated infected mice. Similarly to untreated infected group, fraction C-treated-mice displayed significant weight loss (Fig. 3f). There were no significant differences in haematocrit between the groups (see Additional file 6B).

### Administration of *Agaricus blazei* results in modulation of immune response and protection against brain damage during *Plasmodium berghei* infection

**Fig. 4:**
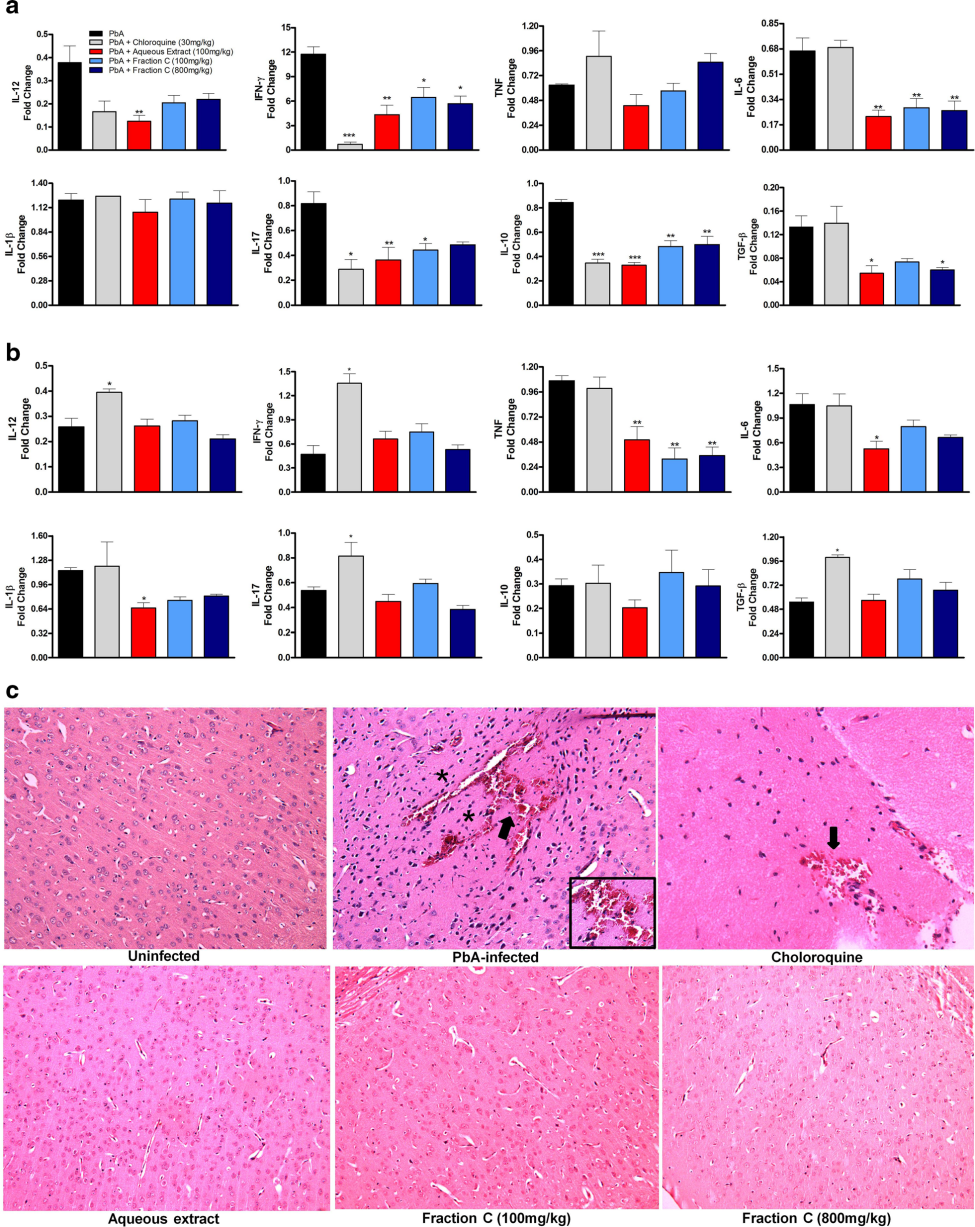
Administration of *Agaricus blazei* modulated cytokine expression in the spleen and brain and protected against CM development. C57BL/6 mice were pre-treated with 100 mg/kg of *A. blazei* extract, Fraction C (100 or 800 mg/kg) or chloroquine (30 mg/kg) 3 days before infection, then infected with 10^5^ parasitized RBCs IP via, and treated until 5 dpi. Production of IL-12, IFN-γ, TNF, IL-6, IL-1β, IL-17, IL-10 and TGF-β in the spleen (**a**) and in the brain (**b**) in response to infection with *P. berghei*, measured at 5 dpi. Cytokines expression was normalized relative to that of uninfected controls for each mouse group. **c** Representative histological (H&E) stained of cerebral cortex of mice, demonstrating severity of cerebral pathology on 5 dpi. It is observed cerebral cortex with normal histological aspect, containing healthy neurons in mice-uninfected and with extensive hemorrhagic area (*arrow*), surrounded by hyperchromatic and condensed neurons (*asterisks*) in *P. berghei*-infected animal. Small haemorrhagic focus (*arrow*) was detected in the brain of mice treated with chloroquine and cerebral parenchyma with normal histological aspect in mice treated with AbM aqueous extract or fraction C. Original magnification ×20. Values are expressed as mean ± SD of 5 mice per group. *p < 0.05, **p < 0.01 and ***p < 0.001 compared to *P. berghei*-infected mice, ANOVA followed Bonferroni’s test.

Infection-associated induction of pro-inflammatory cytokines such as IFN-γ, TNF and IL-1β poses a double-edged sword. Cytokines are essential for control of parasite growth but are also associated with tissue damage both in experimental and clinical settings. In the spleen, the levels of TNF and IL-1β did not change as a result of the administration of the extract. IL-12 production was significantly decreased in mice receiving aqueous extract when compared with untreated infected mice (Fig. 4a). Infected mice that received the aqueous extract or fraction C displayed a significant reduction of IL-6, IL-10, IL-17, IFN-γ and TGF-β levels when compared with untreated infected mice. Similar results were also observed in infected mice that received the chloroquine demonstrating reduced levels of those cytokines, except for IL-6 and TGF-β when compared to untreated-infected mice (Fig. 4a).

There was a significant reduction of TNF, IL-1β and IL-6 levels in the brains of infected mice that received the *A. blazei* when compared to untreated-infected mice. There were no significant differences in IL-10, IL-12, IL-17, IFN-γ and TGF-β levels among the groups treated with *A. blazei* compared with untreated-infected mice (Fig. 4b). Increased levels of IL-12, IL-17, IFN-γ and TGF-β were observed in the brain of infected chloroquine-treated mice when compared with untreated infected mice. Taken together, these data suggest that *A. blazei* modulates cytokine production in the spleen and brain of infected mice (Fig. 4a, b).

Next, was examined whether *A. blazei* aqueous extract or fraction C protected the brain of *P. berghei*-infected mice. Histological examination of brains from infected at 5 dpi revealed necrosis and hemorrhage in the cerebral cortex of untreated infected mice in contrast to *A. blazei*-treated infected mice. In chloroquine-treated mice there were areas of discrete hemorrhage (Fig. 4c).

### Anti-parasitic effect of *Agaricus blazei* in *Plasmodium berghei*

**Fig. 5:**
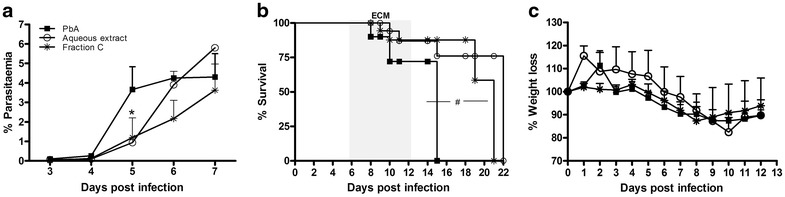
Anti-parasitic effects of extract or fraction C of *Agaricus blazei*. **a** Parasitaemia, **b** survival and **c** body weight of mice infected with untreated pRBCs or treated during 1 h with *A. blazei* water extract or fraction C (500 μg/mL) in vitro. Neurologic signs of CM appeared on days 6–12 (*shaded area*), with death occurring 24–48 h after onset. Parasitaemia and body weight values are expressed as mean ± SD of 4 mice per group. ^#^ *p* < 0.001, log-rank test and *p < 0.05, ANOVA followed Bonferroni’s test.

To evaluate the direct effect of *A. blazei* on the parasite, *P. berghei*-infected red blood cells (RBCs) were pre-treated with *A. blazei* aqueous extract or fraction C for 1 h in vitro, and then was injected (i.p.) these treated RBCs into C57BL/6 mice. Mice injected with untreated-parasitized RBCs developed CM associated with ataxia, loss of grip strength, progressive paralysis, and coma succumbing between 8 and 11 dpi (~80 %)(Fig. 5). However, mice receiving *P. berghei*-infected RBCs treated with *A. blazei* aqueous extract or fraction C displayed significantly parasitaemia reduction, no neurological dysfunction and significant increased survival (Fig. 5a, b). All groups displayed progressive decline in weight (Fig. 5c).

## Discussion

The major findings of the current study can be summarized as follows: (1) *A. blazei* crude aqueous extract is effective in vitro and in vivo against *P. berghei*; (2) the activity resides mainly in a fraction of low MW compounds, denominated fraction C; (3) fraction C delays lethality and protects from tissue damage. Indeed, fraction C is more effective than chloroquine in protecting against cerebral damage caused by the parasite; (4) incubation of the parasite with fraction C prior to infection causes partial protection from disease, suggesting the compound has direct effects on the parasite. Altogether, these findings suggest that molecules derived from *A. blazei* may be useful for the treatment of cerebral malaria.

Herein, it was reported that the administration of *A. blazei* extract to mice infected with *P. berghei* improved the development of CM. Both *A. blazei* aqueous extract and purified fraction C have antioxidant activity, which may be responsible for their anti-parasitic and immunomodulatory effects. It has been demonstrated that *A. blazei* decreased lipid peroxidation in aged rats, demonstrating the capacity of the mixture to possess antioxidant effects in vivo [43]. *A. blazei* fraction C is a mixture of low MW compounds, including phenolic compounds, carbohydrates and peptides (Fig. 3). Other studies demonstrated that phenolic compounds and peptides may have immunomodulatory and/or anti-parasitic activity [13, 44]. It was demonstrated that chemical analysis of *A. blazei* by phytochemical screening indicated the presence of glycoproteins, carbohydrates and tannins [20]. There were three phenolic compounds in *A. blazei*—gallic acid, syringic acid, and pyrogallol. These compounds possess pronounced antioxidant activity [45]. Additional studies will be required to elucidate the precise molecular composition of fraction C that is responsible for anti-parasitic and immunomodulatory activity and whether the antioxidant activity does indeed contribute to the protection observed in vivo.

Oxidative stress has been linked to neurological and cognitive impairment in CM [46]. In the present study, we demonstrated that administration of *A. blazei* aqueous extract or fraction C protected the brain from the damaging effects of this infection. In this regard, previous studies indicated that one activity of this mushroom is related with ROS scavenging and inhibition of lipid peroxidation [4, 12, 47], which may be attributed, at least in part, to the phenolic compounds identified in *A. blazei* [20]. Additionally, previous studies have demonstrated that *A. blazei* treatment was successful in improving the oxidative state of the brain of old rats and was capable of decrease levels of lipid peroxidation (TBARS) of old rats [43]. These results suggest that treatment with *A. blazei* can provide antioxidant activity to the brain in vivo.

It has been reported that antioxidants act by reducing ROS activity and inhibiting lipid peroxidation induced by *Plasmodium* in membrane of parasitized erythrocytes [48], suggesting that the observed anti-malarial effect may be related to the antioxidant activity of fraction C. This effect could be, in part, the result of iron chelation [3]. Anti-malarial drugs such as chloroquine, amodiaquine, primaquine and artemisinin are thought to kill the parasite by the induction of oxidative stress to eliminate the parasite [49]. However, this may also lead to ROS-induced damage to the host [49] as well as drug resistance [50, 51]. In fact, chloroquine treatment, although effective in preventing mortality and reducing parasitaemia, did not prevent protection against brain damage [46, 52]. Therefore, the antioxidant activity of fraction C may be important for the control of CM development in the experimental model of severe malaria.

In fact, the present study demonstrates that the administration of fraction C did not completely eliminate parasitaemia and weight loss despite significantly increasing survival of mice and protecting the brain. This is in agreement with studies by Ibrahim et al. [48] that using the antioxidant also failed to control the parasitaemia in mice infected with *P. berghei*. It is important to note that the elimination of parasitaemia is not always associated with improvement in clinical outcome either in humans or in animal models [29, 53, 54]. Similarly, while the increased production of cytokines in the early infection is important for parasite control it likely contributes to brain damage and cognitive dysfunction associated with CM.

Our results indicate that the administration of *A. blazei* in *P. berghei*-infected mice modified the profile of cytokines produced in the spleen compared to *P. berghei*-infected or chloroquine-treated-infected mice. There was a reduction in the expression of TNF, IL-1β and IL-6 in the brain of *A. blazei* aqueous extract-treated mice and of TNF in *A. blazei* fraction C-treated mice. The induction of these cytokines is associated with the expression of cell adhesion molecules, synthesis of other proinflammatory cytokines, apoptosis and activated phagocytes, all of which have been described in CM [53, 55, 56]. Studies by Lyke et al. [57] and Wunderlich et al. [58], demonstrated that high IL-6 levels were detected in children and mice that developed severe CM. Thus, the reduction levels of TNF, IL-1β and IL-6 levels in *A. blazei*-treated mice could be related to brain protection and increase in survival (Figs. 4b, c, 2b, 3e), observed in the study.

Treatment with chloroquine resulted in an increased production of most proinflammatory cytokines in the brain (IL-12, IFN-γ and IL-17) when compared to untreated-infected and *A. blazei*-treated infected mice. The elevation of TGF-β in chloroquine-treated-infected mice may be associated with two possibilities: (1) inhibit/diminish the proinflammatory response or (2) could be involved with the brain damage. These alterations (elevation of IL-12, IFN-γ, IL-17 and TGF-β) could explain, in part, the histopathologic changes observed in chloroquine-treated mice (Fig. 4c). In agreement, it have been previously reported in clinical and experimental studies a role for TGF-β in malaria severity [59–61]. Taken together, our findings indicate a potential deleterious effect of chloroquine in the CNS not observed with *A. blazei* treatment. Other studies have shown that chloroquine was highly effective in decreasing parasitaemia and preventing mortality, but failed to prevent cognitive and behavioral functions altered in mice subjected to CM. These studies suggest that anti-malarial treatment is not sufficient to protect against the consequences of CM and limiting the inflammatory damage in the brain is necessary in addition to anti-malarials to improve CM outcome [46, 52, 62].

The injection of *P. berghei*-infected RBCs pre-incubated with *A. blazei* aqueous extract or fraction C, was effective in preventing CM. Although this incubation period was brief, it was effective and similar to previous findings with *Leishmania* species [18–20]. This result indicated a direct effect of *A. blazei* on parasite suggesting that it could contribute to control of parasite replication.

## Conclusion

Treatment with the mushroom *A. blazei* is effective in preventing the development of experimental CM and may represent an alternative therapy in the treatment of malaria. Additional studies are needed to identification of compounds isolated from *A. blazei* fraction C responsible for the anti-malarial activity and establish its molecular mechanism of action. Moreover, the efficacy of the combination with other known anti-malarial drugs, and their long-term effect on outcome remains to be fully addressed. These studies may lead to novel therapeutic approaches both in the chemoprophylaxis and treatment of malaria.

## Additional files

**Additional file 1.** Administration of *Agaricus blazei* aqueous extract at dose of 10 mg/kg did not confer protection during *P. berghei* ANKA infection. C57BL/6 mice received 10 mg/kg of *A. blazei* aqueous extract or chloroquine (30 mg/kg) three days before infection, then infected with 10^5^ pRBCs, and treated until 7 dpi. **(A)** Parasitaemia, **(B)** survival and **(C)** body weight loss of control *P. berghei*-infected mice and administered with chloroquine or aqueous extract of the *A. blazei*. Parasitaemia and body weight values are expressed as mean ± SD of 5 mice per group. Log-rank test and ****p* < 0.001, ANOVA followed Bonferroni’s test.
**Additional file 2.** Administration of 800 mg/kg of *Agaricus blazei* lyophilized extract resulted in significant survival of *P. berghei* ANKA-infected mice. **(A)** Parasitaemia and **(B)** survival of C57BL/6 mice that received 80 mg/kg or 800 mg/kg of *A. blazei* lyophilized extract or 30 mg/kg of chloroquine three days before infection, then infected with 10^5^ pRBCs, and treated until 7 dpi. Parasitaemia expressed as mean ± SD of 5 mice per group. **(A)** ANOVA followed Bonferroni’s test was applied and **(B)** ^#^ *p*<0.001 Log-rank test.
**Additional file 3.** Transaminase determinations revealed no hepatic cytotoxic effects during *A. blazei* administration. C57BL/6 mice received 100 mg/kg of *A. blazei* extract, 100 mg/kg or 800 mg/kg of fraction C or 30 mg/kg of chloroquine three days before infection, then infected with 10^5^ parasitized RBCs IP via, and treated until 5 dpi. Transaminase determinations, **(A)** AST and **(B)** ALT were performed at 5 dpi in serum. Values are expressed as mean ± SD of 5 mice per group and ANOVA followed Bonferroni’s test was applied.
**Additional file 4.** There were no differences in parasitaemia and mortality between those mice treated with fraction A and untreated mice during *P. berghei* ANKA infection. **(A)** Parasitaemia, **(B)** survival and **(C)** body weight loss of C57BL/6 mice that received 10 mg/kg of *A. blazei* fraction A or 30 mg/kg of chloroquine three days before infection, then infected with 10^5^ pRBCs, and treated until 7 dpi. Parasitaemia and body weight values are expressed as mean ± SD of 5 mice per group. Log-rank test and **p* < 0.05, ***p* < 0.01 and ****p* < 0.001, ANOVA followed Bonferroni’s test.
**Additional file 5.** Different doses of fraction B were not effective in controlling the *P. berghei* ANKA infection. **(A)** Parasitaemia, **(B)** survival and **(C)** body weight loss of control *P. berghei*-infected mice and administered chloroquine or fraction B (10, 80 and 800 mg/kg). C57BL/6 mice were treated with *A. blazei* or chloroquine three days before infection, then infected with 10^5^ pRBCs, and treated until 7 dpi. Parasitaemia and body weight values are expressed as mean ± SD of 5 mice per group. Log-rank test and **p* < 0.05, ***p* < 0.01 and ****p* < 0.001, ANOVA followed Bonferroni’s test.
**Additional file 6.** Fraction C treated mice did not display clinical signs of CM and significant differences in haematrocrit during *P. berghei* infection. C57BL/6 mice were treated with fraction C of *A. blazei* or chloroquine three days before infection, then infected with 10^5^ pRBCs, and treated until 7 dpi. **(A)** clinical signs of cerebral malaria assessed by the RMCBS score and **(B)** haematocrit assessed on 5^th^ dpi, normalized relative to that of uninfected controls for each mouse group. RMCBS score and haematocrit values were expressed as mean ± SD of 5 mice per group. ****p* < 0.001, ANOVA followed Bonferroni’s test.

## Abbreviations

ECM: experimental cerebral malaria
CM: cerebral malaria
MW: molecular weight
CNS: central nervous system
PbA: *Plasmodium berghei* ANKA
