# Supplementary figures and images for: Effect of mushroom Agaricus blazei on immune response and development of experimental cerebral malaria
Source: Malar J. 2015 Aug 11;14:311. doi: 10.1186/s12936-015-0832-y (PMC4531523; doi:10.1186/s12936-015-0832-y)

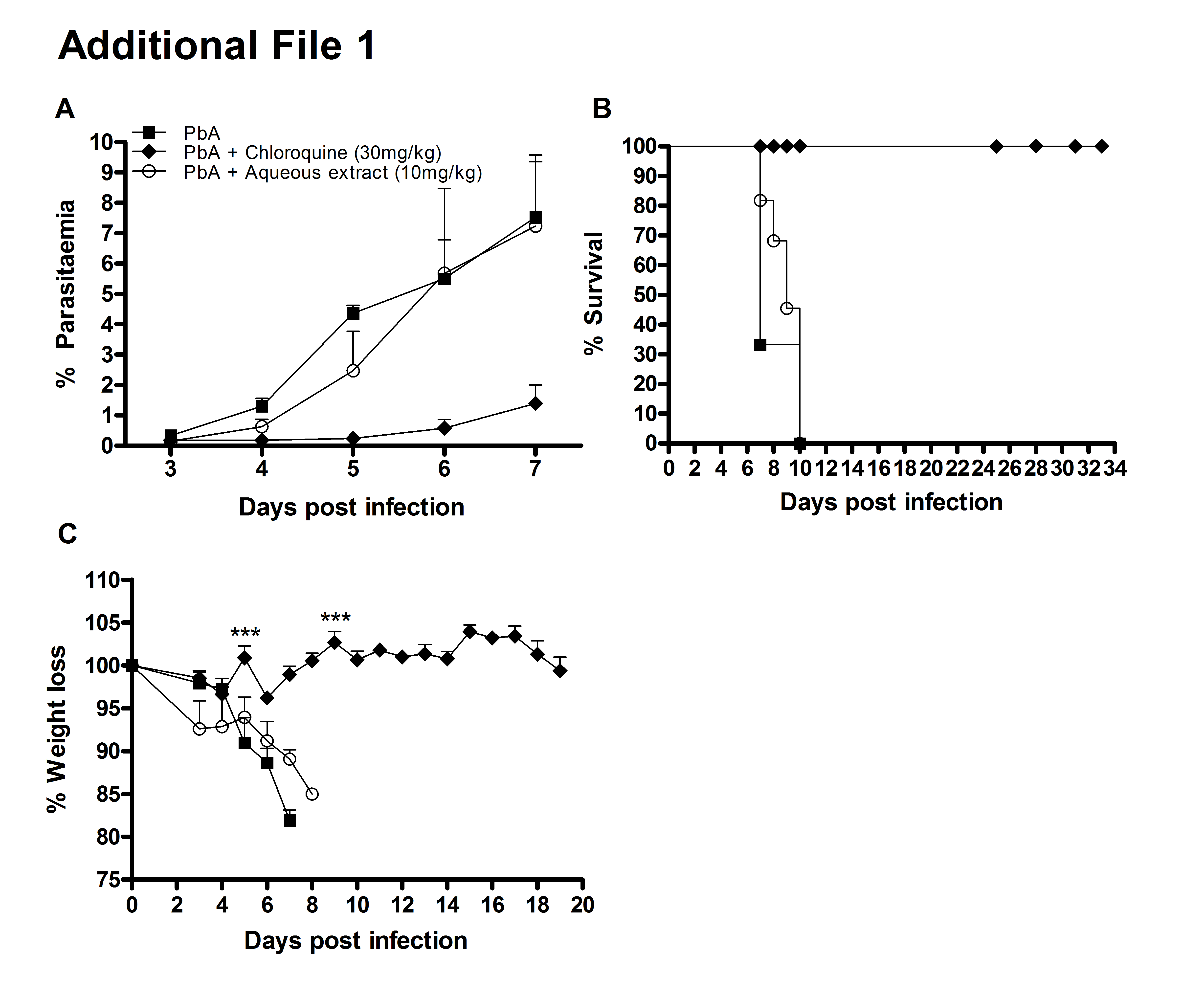

Supplement: Supplementary file 1 — Additional file 1. Administration of Agaricus blazei aqueous extract at dose of 10 mg/kg did not confer protection during P. berghei ANKA infection. C57BL/6 mice received 10 mg/kg of A. blazei aqueous extract or chloroquine (30 mg/kg) three days before infection, then infected with 105 pRBCs, and treated until 7 dpi. (A) Parasitaemia, (B) survival and (C) body weight loss of control P. berghei-infected mice and administered with chloroquine or aqueous extract of the A. blazei. Parasitaemia and body weight values are expressed as mean ± SD of 5 mice per group. Log-rank test and ***p < 0.001, ANOVA followed Bonferroni’s test. [file 12936_2015_832_MOESM1_ESM.tif]

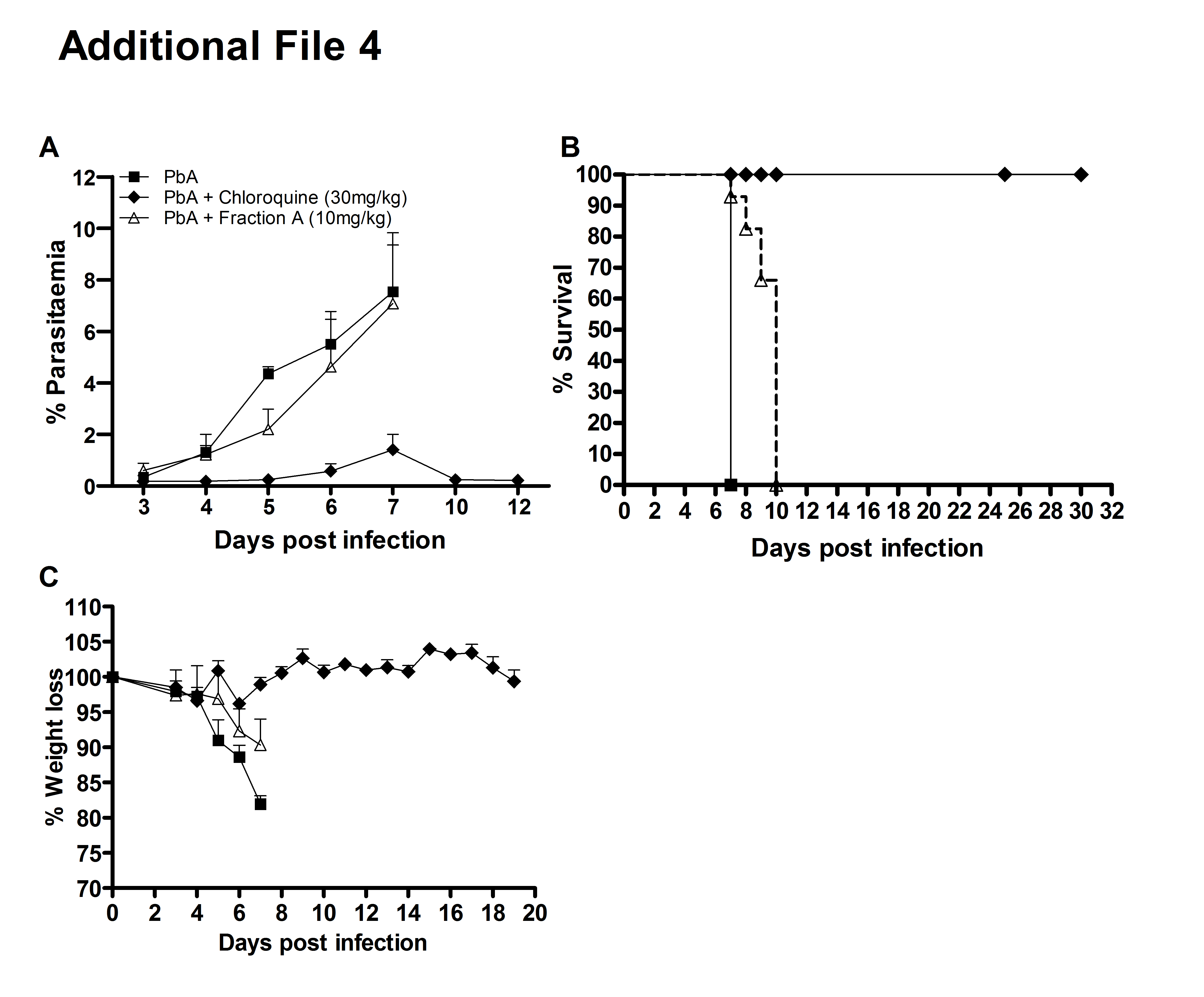

Supplement: Supplementary file 4 — Additional file 4. There were no differences in parasitaemia and mortality between those mice treated with fraction A and untreated mice during P. berghei ANKA infection. (A) Parasitaemia, (B) survival and (C) body weight loss of C57BL/6 mice that received 10 mg/kg of A. blazei fraction A or 30 mg/kg of chloroquine three days before infection, then infected with 105 pRBCs, and treated until 7 dpi. Parasitaemia and body weight values are expressed as mean ± SD of 5 mice per group. Log-rank test and *p < 0.05, **p < 0.01 and ***p < 0.001, ANOVA followed Bonferroni’s test. [file 12936_2015_832_MOESM4_ESM.tif]

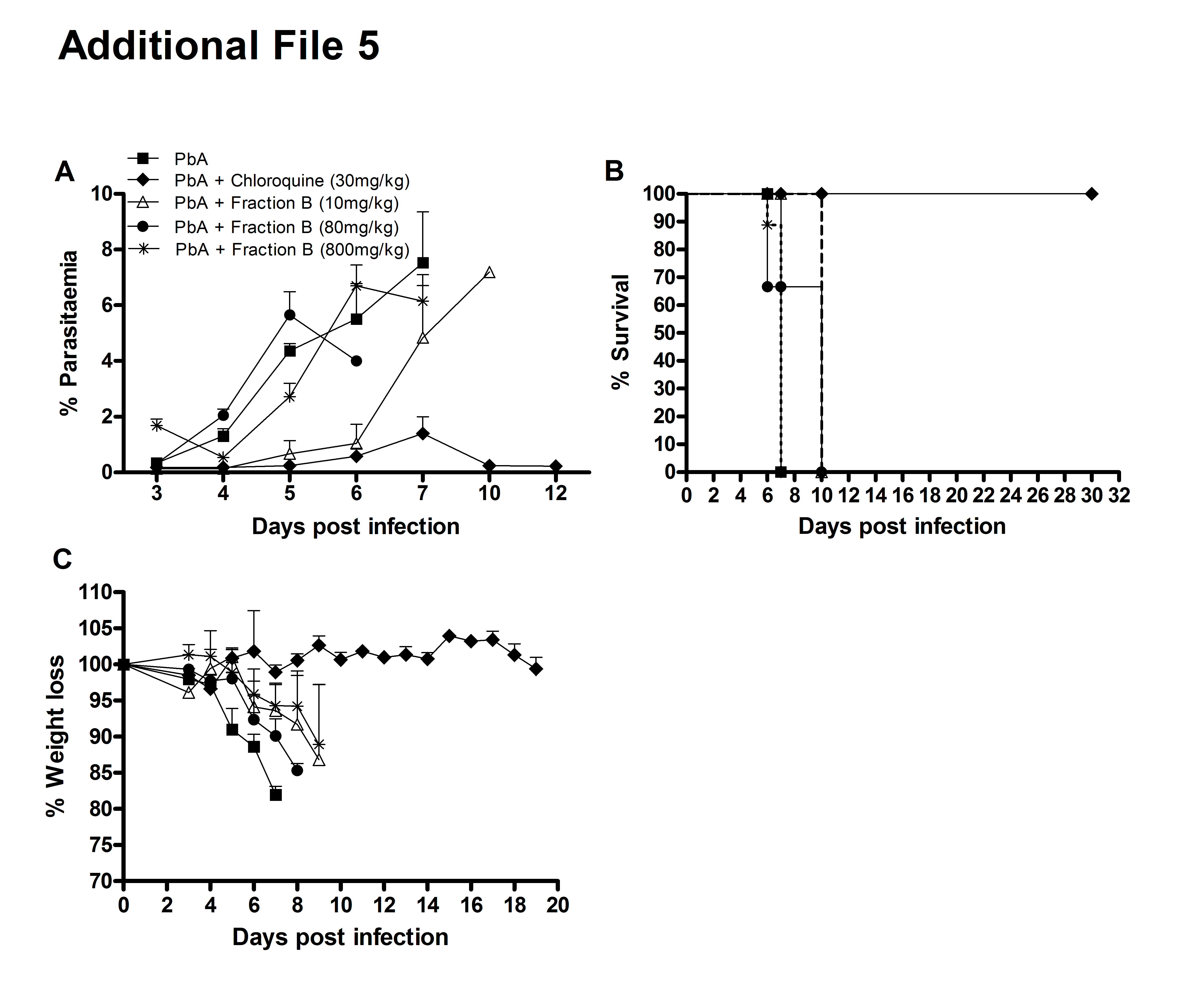

Supplement: Supplementary file 5 — Additional file 5. Different doses of fraction B were not effective in controlling the P. berghei ANKA infection. (A) Parasitaemia, (B) survival and (C) body weight loss of control P. berghei-infected mice and administered chloroquine or fraction B (10, 80 and 800 mg/kg). C57BL/6 mice were treated with A. blazei or chloroquine three days before infection, then infected with 105 pRBCs, and treated until 7 dpi. Parasitaemia and body weight values are expressed as mean ± SD of 5 mice per group. Log-rank test and *p < 0.05, **p < 0.01 and ***p < 0.001, ANOVA followed Bonferroni’s test. [file 12936_2015_832_MOESM5_ESM.tif]
